# Sinus Arrest Associated With a COVID-19 Nasopharyngeal Swab Procedure: A Case Report

**DOI:** 10.7759/cureus.106988

**Published:** 2026-04-13

**Authors:** Robert M Lester, Sabina Paglialunga

**Affiliations:** 1 Cardiac Safety Services, Celerion, Tempe, USA; 2 Scientific Affairs, Celerion, Tempe, USA

**Keywords:** covid-19, electrocardiogram (ecg), holter monitoring, nasopharyngeal swab, trigeminocardiac reflex

## Abstract

During the COVID-19 pandemic, nasal swabs became a routine test for infection. This non-invasive procedure is typically regarded as benign and rarely associated with complications. However, physical stimulation of the nasopharynx and trigeminal nerve can trigger the trigeminocardiac reflex (TCR), a vagally mediated cardioinhibitory response that can lead to sinus arrest. In this study, a clinical trial participant experienced a 3.4 s episode of sinus arrest which was captured on electrocardiogram telemetry during a routine COVID-19 nasal swab test. Diagnosis of the TCR was based upon the plausibility and reversibility criteria, which are hallmarks of this condition. Healthcare providers need to be aware of this phenomenon as well as other potential complications when performing nasopharyngeal swabs.

## Introduction

The SARS-CoV-2 (COVID-19) pandemic was marked by widespread surveillance and screening of individuals worldwide. This screening involved performing either a buccal or a more sensitive nasopharyngeal swab specimen collection for virus detection. Details of the latter procedure are discussed elsewhere, acknowledging that there may be some variability in how the swabbing is performed [1].

It is well established that rhinologic and transsphenoidal procedures involving the nasopharynx may precipitate brainstem-mediated cardioinhibitory vagal responses termed the trigeminocardiac reflex (TCR). These responses are most often secondary to physical or chemical stimulation of any of the three major branches of the trigeminal nerve and, less commonly, the glossopharyngeal nerve or Gasserian ganglion. The vagal-mediated effects may include sinus bradycardia and sinus arrest, syncope, atrioventricular block, gastric hypermotility, and autonomic perturbation in blood pressure, including hypotension and, rarely, hypertension. These responses are usually transient, asymptomatic, self-limiting, and seldom require any medical intervention.

To date, significant neurally-mediated sinus node dysfunction linked to nasopharyngeal swabbing has only been reported in one case, in which a 25-s period of asystole was recorded in a critically ill patient with COVID-19 infection [2]. Although not as profound, we have documented a brief episode of sinus arrest temporally related to nasal swabbing. As such, the purpose of this study is to highlight that a seemingly benign diagnostic procedure may elicit an unanticipated adverse cardiac event for which healthcare professionals should be aware of and prepared to intervene if necessary.

## Case presentation

The subject was a healthy male volunteer in his 30s who was participating in a clinical research protocol. His past medical history was unremarkable, including no proclivity to vagal responses. His physical examination was normal, as were his vital signs, screening laboratory studies, including toxicology assessment, and electrocardiogram.

**Figure 1:**
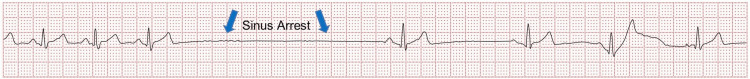
Illustration of sinus arrest presumed secondary to the trigeminocardiac reflex. Sinus arrest occurred during a COVID-19 nasal swab procedure, triggering a presumed TCR. Arrows indicate a 3.4 s period of sinus arrest preceded by sinus tachycardia and followed by transient sinus bradycardia. TCR: trigeminocardiac reflex

The subject was placed on real-time continuous cardiac telemetry monitoring per protocol and had not received study drug prior to undergoing routine COVID-19 screening, which included nasopharyngeal swabbing by a trained and experienced registered nurse. While on telemetry and temporally related to the swabbing procedure, he experienced an asymptomatic 3.4-s period of sinus arrest, as noted in Figure 1.

Sinus rhythm spontaneously and rapidly returned without intervention, and no further episodes of sinus node dysfunction or other dysrhythmias were observed at any time during subsequent telemetry monitoring. Although of scientific interest, there was no effort expended to reproduce the response due to ethical and safety concerns.

## Discussion

Manipulation of the nasopharynx related to craniofacial or transsphenoidal surgical procedures has been well known to result in occasional important and potentially serious adverse cardiovascular responses known as the TCR [3,4]. This phenomenon was first described by Kratschmer et al. in 1870, although it was not formally characterized until 1999 by Schaller et al. based upon their observations during cerebellopontine angle surgery [5,6]. The principal criteria initially proposed for establishing the diagnosis of TCR include three core elements (Table 1) [6].

**Table 1:** Initial criteria for establishing the diagnosis of TCR. TCR: trigeminocardiac reflex

Criteria
Chemical or physical cranial nerve stimulation.
Clear temporal relationship between the stimulus and the response.
Sudden onset of parasympathetic effects, including sinus bradycardia initially defined as a heart rate <60 beats per minute or a 20% decrease from baseline; and/or sympathetic mediated hypotension defined as a 20% reduction in mean arterial pressure.

These diagnostic criteria were updated and refined in 2015 by Meuwly et al. to include the major criteria of plausibility and reversibility, defined below, along with two minor criteria labeled as “repetition and prevention” [7]. To confirm that the TCR is present, both of the following major criteria should be fulfilled: plausibility (the event can be logically explained by the surgical procedure or stimulation of the trigeminal nerve) and reversibility (when the inciting stimulus is stopped, the cardiac event terminates [7,8]).

**Figure 2:**
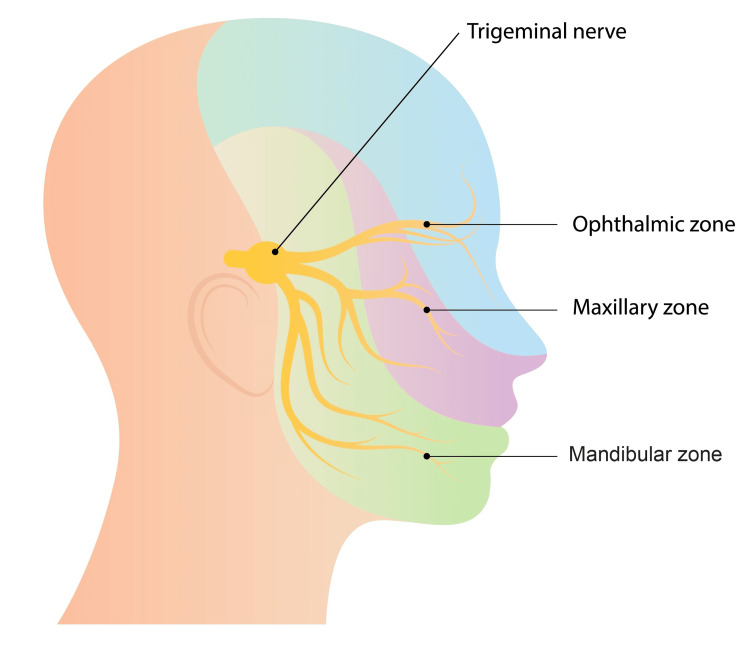
Anatomy of the trigeminal nerve. The image was created by DesignStunn and purchased on Shutterstock.

The pathophysiology of the TCR most commonly relates to stimulation of any of the intracranial (central subtype) or extracranial (peripheral subtype) sensory branches of the trigeminal nerve. Less often, direct excitation of the Gasserian ganglion or glossopharyngeal nerve can also produce identical TCR cardioinhibitory brainstem responses. The trigeminal nerve is the fifth and largest cranial nerve with the following three major branches: the ophthalmic, maxillary, and mandibular branches (Figure 2).

While the maxillary division is predominantly responsible for the TCR, stimulation of any trigeminal nerve branches can send afferent impulses to the brainstem via the Gasserian ganglion and then via an efferent pathway to the vagus nerve, which transmits signals that trigger the TCR. The clinical presentation of the TCR may vary and is dependent upon which branch of the trigeminal nerve is involved. The most common manifestations include sudden onset bradycardia with or without associated hypotension, while syncope, apnea, and gastric hypermotility are less commonly observed [5,9,10].

The present case is unique in that the individual was a healthy male and, by serendipity, happened to be on continuous cardiac telemetry. This is unlike the case report of nasal swab-associated asystole involving a 40-year-old critically ill inpatient infected with COVID-19 [11]. The patient was sedated on mechanical ventilation while receiving multiple medications, and it is not possible to parse out whether any of these factors may have predisposed and contributed to the asystolic event. Our report of transient sinus arrest, although a less pernicious arrhythmia, appears to fulfill the “plausibility and reversibility” criteria for diagnosing the TCR despite the absence of associated hemodynamic findings. Moreover, although it is unlikely, we cannot definitively exclude that our participant may have had pre-existing sinus node disease, which was coincidentally captured during predose telemetry monitoring.

## Conclusions

The true incidence of the TCR related to nasal swabbing is unknown, as individuals are not typically placed on real-time cardiac telemetry or undergo continuous blood pressure monitoring while the sample is being collected. Furthermore, since the episodes are usually asymptomatic and self-limiting, they would not readily be identified unless the cardiac or extracardiac manifestations are profound or warrant therapeutic intervention. Nonetheless, this case underscores that even seemingly innocuous procedures can have unanticipated and potentially serious untoward effects. Finally, although this report is not intended to change nasal swabbing practice, healthcare providers should be aware of this phenomenon when performing nasopharyngeal swabs.
